# Ferroptosis in lymphoma: Emerging mechanisms and a novel therapeutic approach

**DOI:** 10.3389/fgene.2022.1039951

**Published:** 2022-11-03

**Authors:** Qiao Zhou, Ting Li, Qin Qin, Xiaobo Huang, Yi Wang

**Affiliations:** ^1^ Clinical Immunology Translational Medicine Key Laboratory of Sichuan Province, Sichuan Provincial People’s Hospital, University of Electronic Science and Technology of China, Chengdu, China; ^2^ Department of Rheumatology and Immunology, Sichuan Academy of Medical Science and Sichuan Provincial People’s Hospital, University of Electronic Science and Technology of China, Chengdu, China; ^3^ Department of Rheumatology, Wenjiang District People’s Hospital, Chengdu, China; ^4^ School of Medicine, University of Electronic Science and Technology of China, Chengdu, China; ^5^ Department of Critical Care Medicine, Sichuan Academy of Medical Science and Sichuan Provincial People’s Hospital, University of Electronic Science and Technology of China, Chengdu, China

**Keywords:** ferroptosis, iron metabolism, lipid Metabolism, reactive oxygen species (ROS), lymphoma, therapeutic applications, ferroptosis inducer cell death

## Abstract

Unlike apoptosis, necroptosis, autophagy, and pyroptosis, ferroptosis represents a new type of cell death, which is characterized by iron-dependent lipid peroxidation. This process relies largely on the metabolite reactive oxygen species (ROS), phospholipids containing polyunsaturated fatty acids (PUFA-PL), transition metal iron, intra-, and intercellular signaling events, and environmental stress that regulate cellular metabolism and ROS levels. Recent studies show that ferroptosis plays an important role in tumorigenesis, tumor development, and the treatment of hematological malignancies, including lymphoma. Despite the constant emergence of new drugs, the differences in morphological features, immunophenotypes, biological patterns, rates of onset, and response to treatment in lymphoma pose major therapeutic challenges. Since lymphoma is associated with ferroptosis and shows sensitivity towards it, targeting the potential regulatory factors may regulate lymphoma progression. This has emerged as a research hotspot. This review summarizes the current knowledge on ferroptosis induction and resistance mechanisms, their roles and mechanistic details of ferroptosis in lymphoma suppression and immunity, and finally the treatment strategies for lymphoma by targeting ferroptosis.

## 1 Introduction

Cell death, including apoptosis (Nagata, 2018), NETosis (Segura et al., 2020), necroptosis (Guo et al., 2019), autophagy (Bonam et al., 2018), pyroptosis (Fu et al., 2017), and cuproptosis (Tsvetkov et al., 2022), is an important physiological process that maintains the integrity by regulating organismal metabolism and removing excess or damaged cells.

Ferroptosis, unlike other forms, is a new type of programmed cell death that shows iron-dependence (Li et al., 2020). Several tumor suppressors, including p53 and BAP1, promote ferroptosis by inhibiting cystine uptake, confirming that ferroptosis is a natural barrier against tumor development (Toyokuni et al., 2017; Zhang et al., 2018). Moreover, tumor cells are more prone to ferroptosis due to their unique metabolic program and high load of reactive oxygen species (ROS) (Chen et al., 2020a), rendering ferroptosis inducers as potential targets of cancer therapy, especially against some of the most drug-resistant and aggressive tumors (Hangauer et al., 2017; Viswanathan et al., 2017). Non-Hodgkin’s lymphomas are clinically heterogeneous, and different subtypes show varying prognoses; even in the same type, some cases are curable with combination therapy, while for the remaining, treatment is difficult and patients exhibit poor prognosis (Chapuy et al., 2018). Diffuse large B cell lymphoma (DLBCL), adult T-cell leukemia/lymphoma (ATLL), and Burkitt’s lymphoma (BL) are particularly sensitive to cell death by ferroptosis as shown in recent studies (Yang et al., 2014; Mancuso et al., 2021; Chen et al., 2022). Thus, treatment with ferroptosis inducers may be a promising therapeutic strategy for lymphoma in the foreseeable future.

## 2 Mechanisms underlying ferroptosis

Ferroptosis is driven by increased cellular iron load and is characterized by the loss in membrane integrity and changes in mitochondrial ultrastructure, including reduced mitochondrial volume, increased mitochondrial bilayer membrane density, decreased mitochondrial cristae, and ruptured mitochondrial membrane (Doll et al., 2017; Du et al., 2022; Zhou et al., 2022). Ferroptosis can be induced through the interaction of iron regulation, lipid metabolism, and ROS biology, and research in these fields is expected to deepen the mechanistic understanding, biological significance, and clinical therapeutic relevance of ferroptosis (Table 1). However, to date, our understanding of ferroptosis remains incomplete, and the molecular mechanisms that trigger ferroptosis and its selective regulation under certain circumstances, are largely unclear (Davidson and Wood, 2020).

**TABLE 1:** Inducers and inhibitors of ferroptosis.

	Drugs or pathways	Mechanisms
Inducer	ATM, ferritinophagy	Regulate ferritin abundance
	FINO2	Oxidize ferrous iron and lipidome, inactivate GPX4
	FIN56	Degrade GPX4 and deplete antioxidant CoQ10
	RSL3, DPI7	inhibit GPX4
	HO-1	Supplement iron
	Erastin	Inhibit system Xc- and degrade GPX4
	CDO1	Deplete cysteine
	P53 protein	Supress SLC7A11
	MDR1	Cause efflux of GSH
Inhibitor	Ferroportin, MVBs	Deplete labile iron
	HSPB1	Inhibit TRF1
	FSP1/CoQ10/NADPH	Inhibit phospholipid peroxidation
	DHODH	Reduce mithchondrial CoQ10
	GCH1/BH4	Prevetn lipid peroxdation, deplete PUFA-PL
	Transsulfuration way	Produce cysteine from methionine
	mTOR	Increase GPX4 synthesiss
	P53-P21 pathway	Slow depletion of intracellular GSH and reduce accumulation of ROS

ATM, serine/threonine kinase; *RSL3,* RAS-selective-lethal-3; *HO-1*, Heme oxygenase-1; *CDO1,* cysteine dioxygenase1; *MDR1,* multidrug resistance gene; *MVBs*, multivesicular bodies; *HSPB1,* Heat shock protein beta-1; *DHODH*, dihydroorotate dehydrogenase; *GCH/BH4*, GTP cyclohydrolase 1/tetrahydrobiopterin; *mTOR*, mechanistic target of the rapamycin.

### 2.1 Role of iron metabolism in ferroptosis

Fe2+ first binds to transferrin (TF) in the intestine and is subsequently absorbed by intestinal mucosal cells, wherein Fe2+ is oxidized to Fe3+ by ceruloplasmin and other ferroxidases (Cronin et al., 2019). Fe^3+^ enters the capillaries and then binds to TF, which in turn binds to the TF receptor 1 (TfR1) and is endocytosed into cells (Pandrangi et al., 2022). Fe^3+^ is then reduced to Fe2+ by the metalloreductase, six-transmembrane epithelial antigen of the prostate (STEAP3), and subsequently, Fe^2+^ is released into the labile iron pool (LIP) *via* divalent metal transporter one or stored as ferritin, the iron-storage protein (Li et al., 2020). Excessive Fe2+ is oxidized to Fe^3+^ by the cellular iron exporter, ferroportin (FPN) (Li et al., 2021). There are two major mechanisms by which iron ions regulate ferroptosis. First, Fe^2+^ can mediate the Fenton reaction to generate excess ROS, which subsequently reacts with polyunsaturated fatty acids (PUFAs) to promote lipid peroxidation, ultimately leading to ferroptosis (Shah et al., 2018). Second, some enzymes that mediate the formation of lipid hydroperoxides for the Fenton reaction require Fe^2+^ as a cofactor, including arachidonate lipoxygenases (ALOXs) (Li et al., 2021). The regulation of the iron-storage protein, ferritin, *via* ferritinophagy (Stockwell, 2022) and the serine/threonine kinase mutated in Ataxia-Telangiectasia (ATM) (Chen et al., 2020a) control the size of the LIP, which promotes labile iron accumulation and regulates cellular sensitivity to ferroptosis (Chen et al., 2020a; Stockwell, 2022). Ferroptosis-inducer-1,2-dioxolane (FINO2) induces ferroptosis by oxidizing Fe^2+^ to Fe^3+^ through the Fenton reaction to generate alkoxyl radical which initiates lipid peroxidation. Moreover, FINO2 can indirectly inactivate GPX4 by depleting glutathione (GSH) (Abrams et al., 2016). Fe^2+^ from the LIP binds to GSH(Patel et al., 2019) and its subsequent depletion promotes the availability of labile iron, which further mobilizes Fe^2+^ for the Fenton reaction, thereby promoting lipid peroxidation and ferroptosis (Patel et al., 2019). Heme oxygenase-1 (HO-1) can induce ferroptosis by promoting the accumulation of intracellular ferrous ions (Jing et al., 2022). Ferroportin and prominin-2-mediated ferritin-containing multivesicular bodies can deplete the LIP and induce resistance to ferroptosis (Brown et al., 2019). Overexpression of heat shock protein beta-1 can inhibit ferroptosis by downregulating TfR1-mediated iron uptake by stabilizing the F-actin cytoskeleton (Ren et al., 2020). Consequently, silencing the TFRC gene encoding TfR1 protein can inhibit ferroptosis (Song et al., 2021).

### 2.2 Lipid metabolism in ferroptosis

Ferroptosis is mainly driven by the peroxidation of specific membrane lipids. PUFAs are highly susceptible to ROS-induced peroxidation and are crucial to ferroptosis (Conrad et al., 2018). Free PUFAs cannot induce ferroptosis. After incorporation into membrane lipids, including phospholipids (PLs), the excessive accumulation of oxidized (PUFA-PLs) can contribute to ferroptosis (Xu et al., 2021). Lysophosphatidylcholine acyltransferase 3 and acyl-coenzyme A synthetase long-chain family member 4 (ACSL4) are involved in activating and incorporating PUFAs into PLs (Brown et al., 2019). Other ACSL enzymes, including ACSL1, are required to exert the pro-ferroptosis activity of conjugated linolenic acids (Beatty et al., 2021). Moreover, monounsaturated fatty acids (MUFAs), including oleic acid and palmitoleic acid, are not susceptible to peroxidation due to a lack of a bisallyl moiety (Magtanong et al., 2019). In turn, they require ACSL3 to displace the PUFA component from PUFA-PLs, thus exerting anti-ferroptosis effects (Magtanong et al., 2019). Activation of the liver kinase B1-AMP-activated protein kinase axis also plays a protective role against ferroptosis by limiting PUFA biosynthesis by inhibiting the phosphorylation of acetyl-CoA carboxylase (ACC) (Lee et al., 2020).

### 2.3 ROS accumulation in ferroptosis

Generation of ROS is a result of Fenton reactions and its accumulation can induce ferroptosis (Tang et al., 2021). The cytoplasmic and mitochondrial cystine/cysteine/GSH/GPX4 axis is a central regulator to protect cells from ferroptosis (Jiang et al., 2021). System Xc-is a heterodimeric 12-pass transmembrane cystine/glutamate antiporter comprising SLC3A2 and SLC7A11 subunits, which import cystine that is reduced to cysteine in the cell (Sato et al., 2018). Cysteine and glutamate are substrates for the biosynthesis of GSH, and GPX4 is a selenoprotein acting as a GSH-dependent peroxidase against lipid peroxidation by reducing the toxic lipid peroxide PL-PUFA-OOH to non-toxic PUFA-PL-OH (Ursini and Maiorino, 2020).

Substances that induce ferroptosis through the cystine/cysteine/GSH/GPX4 axis can be divided into four categories. Erastin is the prototype ferroptosis inducer that reduces cellular cystine uptake by directly inhibiting system Xc- and promotes the degradation of GPX4 by enhancing chaperone-mediated autophagy (Wu et al., 2019). Rat sarcoma-selective-lethal-3 (RSL3) and 5,6-dihydro-2H-pyrano [3,2-g]indolizine-7 can induce ferroptosis by directly inhibiting the activity of GPX4 (7). Ferroptosis-Inducer-56 (FIN56) can induce ferroptosis either by promoting GPX4 degradation or depleting endogenous antioxidant coenzyme Q10 (COQ10) (Liang et al., 2019). FINO2, as discussed above, can also initiate ferroptosis by indirectly inhibiting GPX4 (42). Other new molecules that induce ferroptosis have been recently discovered. The multidrug resistance gene, MDR1, increases cellular sensitivity to ferroptosis through GSH efflux (Cao et al., 2019), and the iron metalloenzyme, cysteine dioxygenase1, drives cell susceptibility to ferroptosis by depleting cysteine, which in turn reduces GSH (Hao et al., 2017).

GPX4 plays an important role in maintaining intracellular redox homeostasis and is the primary enzyme that inhibits ferroptosis. However, three other GPX4-independent systems also play a role in suppressing ferroptosis. The ferroptosis suppressor protein 1 (FSP1)/CoQ10/nicotinamide adenine dinucleotide phosphate (NADPH) pathway regulates ferroptosis independent of GPX4 and GSH. FSP1 catalyzes the formation of reduced CoQ10 in an NADPH-dependent manner, thereby reducing lipid free radicals and ultimately inhibiting lipid peroxidation and ferroptosis (Bersuker et al., 2019). Dihydroorotate dehydrogenase inhibits mitochondrial lipid peroxidation and ferroptosis by reducing mitochondrial CoQ10 to CoQH2 (Mao et al., 2021). The GTP cyclohydrolase 1/tetrahydrobiopterin axis also inhibits ferroptosis by reducing CoQ10 against lipid peroxidation and depleting PUFA-PLs (Kraft et al., 2020). Furthermore, several negative regulators of ferroptosis are known. The antioxidant transcription factor, NRF2, suppresses ferroptosis by regulating the expression of genes related to GSH biosynthesis, NADPH regeneration, and iron homeostasis (Song and Long, 2020). Trans-sulfuration produces cystine from methionine, providing more substrates for GSH and inhibiting ferroptosis (Zhang et al., 2022). Activation of the recombination activating gene (Rag)- the mechanistic target of rapamycin complex 1 (mTORC1)-eukaryotic initiation factor 4E (eIF4E)-binding proteins (4EBPs) signaling axis induces resistance to ferroptosis by increasing GPX4 synthesis (Zhang et al., 2021) and the phosphatidylinositol 3-kinase-alpha serine/threonine-protein kinase-the mechanistic target of rapamycin pathway can suppress ferroptosis by increasing sterol regulatory element-binding protein-mediated lipogenesis (Yi et al., 2020).

### 2.4 p53 in ferroptosis

p53 can inhibit the expression of SLC7A11 at the transcriptional level and subsequently reduce the biosynthesis of GSH, thus inducing ferroptosis and suppressing tumor growth (Hu et al., 2022). In 2019, Gu Wei *et al.* demonstrated that the p53-SLC7A11 axis can also promote ferroptosis in a GSH-independent manner (Liu and Gu, 2022). ALOX12, a lipoxygenase, oxidizes PUFA and induces cellular ferroptosis. p53 induces ALOX12 activity by repressing SLC7A11 expression as the latter can directly bind to the former, thereby limiting its functions (Liu and Gu, 2022). p53 induces SAT1 expression, thus promoting the function of ALOX15 and inducing ferroptosis (Liu and Gu, 2022). Recently, a new target gene of p53, independent phospholipase A2beta, was found to suppress ferroptosis by cleaving peroxidized lipids and releasing PUFAs from membrane PLs (Chen et al., 2021a). However, p53 also inhibits ferroptosis under certain circumstances, a process requiring the involvement of P21, a p53 transcriptional target that can inhibit cell cycle progression, thereby converting part of the raw materials necessary for the synthesis of nucleic acids to those required for NADPH and GSH and inhibiting ferroptosis (Tarangelo et al., 2018). Therefore, p53 may regulate ferroptosis through distinct mechanisms which need further investigation.

## 3 Potential role of ferroptosis in lymphoma

DLBCL is the most common type of adult non-Hodgkin’s lymphoma (NHL), accounting for nearly 30% of the cases of adult NHL wordwide (Beham-Schmid, 2017). DLBCL cell lines are susceptible to ferroptosis induced by system Xc-inhibitors because of their inability to use the trans-sulfuration pathway to convert methionine to cysteine (Gout et al., 2001; Sun et al., 2014). Ferroptosis slows down tumor growth in a DLBCL xenograft model (Deng et al., 2019). Yuko Kinowaki et al. (2018) assessed the expression of GPX4 by immunohistochemistry and found that the GPX4-positive group showed poor overall survival relative to the GPX4-negative group, indicating that GPX4 overexpression is an independent prognostic predictor of adverse prognosis in DLBCL. Moreover, the activation of ferroptosis participates in p53-mediated cell cycle arrest and cell death in B-cell lymphomas (Li et al., 2016). Hydroxyacyl-CoA dehydrogenase (HADHA) is involved in fatty acid beta-oxidation (FAO) and is overexpressed in high-grade lymphoma. Its overexpression indicates a poor prognosis in DLBCL(62). Recently, some studies have attempted to identify ferroptosis-related genes in DLBCL and elucidate a method to predict the prognosis of these patients, along with novel treatment strategies. Huan Chen et al. (2021b) performed a systematic study and identified a ferroptosis-related gene signature comprising 8 genes that could be used to divide DLBCL patients into high- and low-risk groups. Moreover, Junmei Weng *et al.* built a risk score model related to ferroptosis with 11 genes and revealed that the high-risk score group showed resistance to ibrutinib treatment, while ferroptosis inducer, acetaminophen, inhibited the expression of the high-risk genes in the DLBCL cell lines (Weng et al., 2022). Julie Devin et al. (2022) built the iron score-related model to identify patients with DLBCL showing poor prognosis who might benefit from treatment targeted to maintaining iron homeostasis. All these studies suggest that therapeutic drugs targeting ferroptosis might contribute to alleviating DLBCL.

Despite limited information, other types of lymphoma that have been studied in the light of ferroptosis, include adult T-cell leukemia/lymphoma, BL, and natural killer/T cell lymphoma (NKTCL) (Du et al., 2022). These lymphomas are highly invasive and treatment options are limited especially in patients older than 60 years. Ferroptosis inducers, especially artesunate, alleviate these types of lymphoma through different mechanisms (Wang et al., 2019; Ishikawa et al., 2020; He et al., 2022), thus providing perspectives for the development of novel therapeutic strategies. However, unlike DLBCL, basic research for elucidating the mechanism of ferroptosis on tumorigenesis of these lymphomas is lacking.

## 4 Therapeutic applications

**FIGURE 1:**
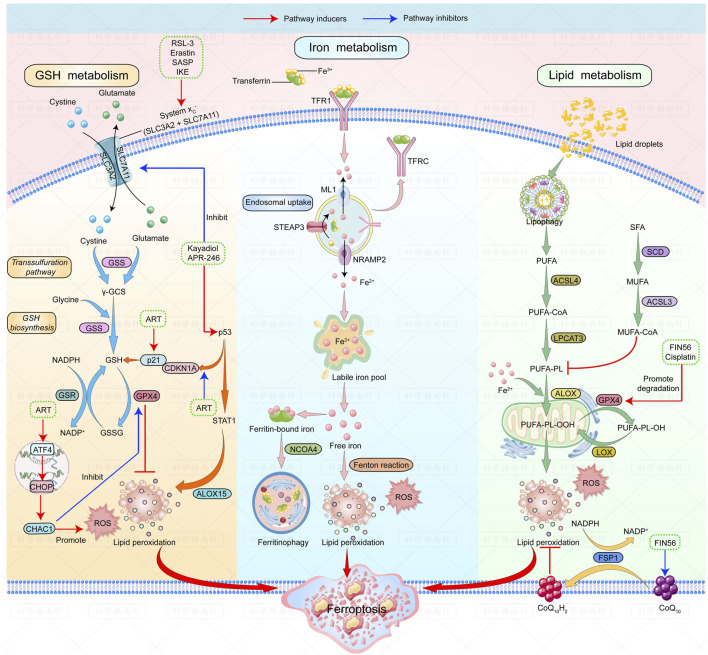
| Mechanisms of ferroptosis and ferroptosis inducers in lymphoma. The figure shows the regulatory pathways of ferroptosis can be divided into three categories. The first one is regulated by GSH/GPX4 pathway. The second one is the iron metabolism pathway that regulates the labile iron pool. The third category is the lipid metabolism pathways. Ferroptosis inducers that have been studied in lymphoma are listed: RSL-3, erastin, SASP and IKE can induce ferroptosis by inhibiting system Xc-; Kayadiol and APR-246 induce ferroptosis by both inhibiting system Xc- and promoting P53-P21 pathway; ART could induce ferroptosis by activating the ATF4-CHOP-CHAC1 pathway or by increasing p21 expression. Fin56 induces ferroptosis by promoting degradation of GPX4 and depleting endogenous antioxidant coenzyme Q10. Abbreviations: RSL-3, RAS-selective-lethal-3; SASP, safasalazine; IKE, Imidazole-ketone-erastin; ART, Artesunate.

Several ferroptosis inducers against lymphoma have shown good outcomes in animal models. A summary of ferroptosis inducers against lymphomas is shown in Figure 1.

### 4.1 Drugs targeting system Xc-

GSH metabolic pathways are classical targets of ferroptosis inducers. Erastin and its analog, RSL3, and sulfasalazine (SASP), have been widely applied as classical ferroptosis-inducing agents by targeting system Xc- and GPX4 (Xiao et al., 2015; Zhang et al., 2020). Imidazole-ketone-erastin (IKE), an improved erastin analog, can inhibit system Xc-at low concentrations (Ye et al., 2020). Zhang et al. (2019) demonstrated that IKE prevented tumor progression by inhibiting system Xc-in a DLBCL xenograft model through GSH depletion and lipid peroxidation. Erastin and RSL3-termed ferroptosis inducers can inhibit tumor growth in two DLBCL cell lines (Yang et al., 2014). In rats treated with sulfasalazine (SASP), a system Xc-inhibitor, the transplanted lymphoma growth shows marked reduction (Gout et al., 2003). As lymphoid cells cannot synthesize cysteine and since their growth is dependent on cysteine uptake from the microenvironment, SASP exerts inhibitory effects of cysteine secretion by somatic cells, causing cysteine starvation in lymphoma cells and inducing ferroptosis *in vivo* (Gout et al., 2001).

Since GSH metabolism is well understood, it is easy to speculate that the above drugs should be among the most promising ferroptosis inducers but more *in vivo* experiments are needed to validate their efficacy and safety in the future.

### 4.2 Drugs targeting p53-mediated ferroptosis

Drugs targeting the p53-mediated ferroptosis pathway, including eprenetapopt (or APR-246) and kayadiol, are new research hotspots. APR-246 reactivates the transcriptional activity of the mutant p53 by promoting its binding to target genes, thereby showing efficacy in p53-mutated tumors (Cluzeau et al., 2021; Mishra et al., 2022). APR-246 reactivates the transcriptional activity of mutant p53 by promoting their binding to its target genes and is effective in p53-mutated tumors (Cluzeau et al., 2021; Mishra et al., 2022). APR-246 can induce tumor protein p53 TP53-mutation-mediated cell death in DLBCL through ferroptosis by p53-dependent ferritinophagy (Hong et al., 2022). APR-246 can also induce ferroptosis in a p53-independent manner by binding to GSH or inhibiting antioxidant enzymes in acute myeloid leukemia but this has not been validated in lymphomas yet (Birsen et al., 2022). Thus, APR-246 may be a promising new therapeutic drug for DLBCL patients.

Kayadiol, a diterpenoid, shows a strong inhibitory effect on extranodal NKTCL cells. Kayadiol treatment triggers significant ferroptosis events through the p53-mediated pathway, including ROS accumulation and GSH depletion and reducing the expressions of SLC7A11 and GPX4. Moreover, Kayadiol also promotes the phosphorylation of p53, thus upregulating its protein expression. Hence, Kayadiol can be used as an alternative for NK/T cell lymphoma treatment, especially in cases of failure of initial therapeutic strategies (He et al., 2022).

Ferroptosis inducers involved in the p53-mediated pathway have been recently developed, and APR-246 has been studied in different types of hematologic malignancies, showing good results in clinical trials (Gout et al., 2003). It is expected that drugs targeting the p53 pathway may soon enter clinical trials for lymphoma.

### 4.3 Other ferroptosis inducers

Other types of drugs, including artesunate (ART), the FAO inhibitor, and dimethyl fumarate, can promote ferroptosis through different mechanisms. ART, a widely used antimalarial compound exerting cytotoxicity, induces ferroptosis in different types of lymphomas (Wang et al., 2019; Ishikawa et al., 2020; Chen et al., 2021c). By inducing lysosomal degradation of ferritin, ART increases intracellular labile iron and ROS levels, rendering cells sensitive to ferroptosis (Li, 2012; Chen et al., 2020b). Moreover, ART can inhibit the activation of signal transducer and activator of transcription 3 (STAT3), thereby downregulating the expression of GPX4, finally leading to ferroptosis (Chen et al., 2021c). Ning Wang et al. (2019) showed that ART could induce ferroptosis by activating the transcription factor 4-CEBP-homologous protein-cation transport regulator-like protein 1 (ATF4-CHOP-CHAC1) pathway, thus inhibiting GPX4 expression in BL cell lines. As mentioned above, the upregulation of fatty acid beta-oxidation is observed in high-grade lymphoma (Yamamoto et al., 2020). Inhibition of HADHA may disrupt the balance between saturated and unsaturated fatty acids that are subsequently supplied to PLs, in turn promoting the accumulation of polyunsaturated fatty acids in the cell membrane along with ferroptosis (Zhou et al., 2012a; Zhou et al., 2012b). Treatment with the FAO inhibitor, ranolazine, increases cell death in DLBCL (Sekine et al., 2022). Finally, dimethyl fumarate (DMF) potently and rapidly depletes GSH by inducing the succination of cysteine residues and induces lipid peroxidation, subsequently inducing ferroptosis, particularly in DLBCL (Schmitt et al., 2021).

With the continued and improved understanding of the mechanisms underlying ferroptosis, targeting different pathways to induce ferroptosis in lymphomas is emerging as a promising new option that has been validated in animal and *in vitro* experiments. Follow-up studies should focus on the effects and safety of these drugs in humans.

## 5 Conclusion and perspectives

Ferroptosis is an iron-dependent form of programmed cell death, a new direction for future research. It is regulated by several cellular metabolic pathways such as redox homeostasis, iron metabolism, mitochondrial activity, lipid, and amino acid metabolism, and ROS accumulation, as described above. Novel pathways such as p53-mediated signaling also play a complicated role in regulating of ferroptosis. Presumably, the p53-p21 pathway is activated to protect cells from damage when the external stimuli are small. On the contrary, other p53-mediated pathways are initiated to destroy cells *via* ferroptosis as stimuli exceed the threshold. Several non-classical regulatory pathways associated with ferroptosis are emerging such as mitochondrial VDACs (Skonieczna et al., 2017) and FSP1(45)-induced ferroptosis. Moreover, other kinds of programmed cell death such as autophagy can cause changes in ferritin levels, subsequently increasing the labile iron pool and inducing ferroptosis (Hou et al., 2016). Therefore, crosstalk between ferroptosis and other cell death modes exists but the exact underlying mechanisms remain elusive.

Ferroptosis was proposed for the first time in 2012, and since, it has emerged as an attractive target in tumor biology and cancer therapy. Certain types of tumors are more prone to ferroptosis due to their unique metabolic characteristics and high load of ROS, making ferroptosis a promising candidate for targeted therapy. The sensitivity of lymphoma to ferroptosis can be increased by drugs that regulate intracellular ROS production, iron metabolism, GPX4 levels, and other molecules. As most evidence has been obtained from animal models, there remains a long way toward the clinical application of ferroptosis inducers in lymphoma treatment. More research is needed in the future to uncover all the underlying mechanisms of ferroptosis in lymphoma.

The figure shows the regulatory pathways involved in ferroptosis. There are three main types of mechanisms involved in regulating ferroptosis: first, the regulation by the GSH/GPX4 pathway. The second is the iron metabolism pathway that regulates the labile iron pool. The third includes pathways related to lipid metabolism that affect lipid regulation. Ferroptosis inducers studied in lymphoma are as follows: RSL-3, Erastin, SASP, and IKE can induce ferroptosis by inhibiting system Xc-; Kayadiol and APR-246 induce ferroptosis by both inhibiting system Xc- and promoting the p53-p21 pathway; ART can induce ferroptosis by activating the ATF4-CHOP-CHAC1 pathway or by increasing the expression of P21. FIN56 induces ferroptosis by accelerating GPX4 degradation and depleting the endogenous antioxidant coenzyme, Q10.

## Glossary

ART: Artesunate
ACSL3: Acyl-coenzyme A synthetase long-chain family member 3
ACSL4: Acyl-coenzyme A synthetase long-chain family member 4
ALOX: Arachidonate lipoxygenase
ALOX15: Arachidonate lipoxygenase 15
ATF4-CHOP-CHAC1: Activating transcription factor 4-CEBP-homologous protein-cation transport regulator-like protein 1
CoQ10: Coenzyme Q10
CoQ10H2: Reduced coenzyme Q10
FIN56: Ferroptosis-inducer-56
FSP1: Ferroptosis suppressor protein 1
GPX4: Glutathione peroxidase 4
GSH: Glutathione
GSR: Glutathione reductase
GSS: Glutathione synthetase
GSSG: Oxidized glutathione
IKE: Imidazole-ketone-erastin
LIP: Labile iron pool
LOX: Lipoxygenase
LPCAT3: Lysophosphatidylcholine acyltransferase 3
ML1: Mucolipin 1
MUFA: Monounsaturated fatty acids
MUFA-COA: Monounsaturated fatty acids-coenzyme A
NADPH: Nicotinamide adenine dinucleotide phosphate (NADPH)
NCOA4: Nuclear receptor activator 4
NRAMP2: Natural resistance-associated macrophage protein 2
PUFA: Polyunsaturated fatty acids
PUFA-PL: Polyunsaturated fatty acids of phospholipids
γ-GCS: γ-glutamyl-cysteine synthetase
RSL-3: Rat sarcoma-selective-lethal-3
ROS: Reactive oxygen species;
STAT1: Signal transducer and activator of transcription 1
SASP: Safasalazine
SCD: Stearoyl-CoA desaturase
SFA: Saturated fatty acids
STEAP3: Six-transmembrane epithelial antigen of prostate 3
TFR1: Transferrin receptor 1
TFRC: Transferrin receptor
